# Effects of the Higashi-Nihon Earthquake: Posttraumatic Stress, Psychological Changes, and Cortisol Levels of Survivors

**DOI:** 10.1371/journal.pone.0034612

**Published:** 2012-04-25

**Authors:** Yuka Kotozaki, Ryuta Kawashima

**Affiliations:** Smart Ageing International Research Centre, Institute of Development, Aging and Cancer, Tohoku University, Sendai, Japan; Postgraduate Medical Institute & Hull York Medical School -, University of Hull, United Kingdom

## Abstract

On March 11, 2011, the Pacific side of Japan’s northeast was devastated by an earthquake and tsunami. For years, many researchers have been working on ways of examining the psychological effects of earthquakes on survivors in disaster areas who have experienced aftershocks, catastrophic fires, and other damage caused by the earthquake**.** The goal of this study is to examine scores on psychological measures and salivary cortisol level in these individuals both before and three months after the earthquake. The participants had been measured for these variables before the earthquake. After the earthquake, we carried out PTSD screening using CAPS for participants for another experiment, and then again conducted the aforementioned tests. We collected saliva samples from all survivors. Our results show that social relationship scores on the WHO-QOL26, negative mood scores of the WHO-SUBI, total GHQ score, POMS confusion scores, and CMI emotional status score after the earthquake showed scores indicating significantly decreased compared to before the earthquake. On the other hand, salivary cortisol levels after the earthquake was significantly increased compared to before the earthquake. Moreover, the result of a multiple regression analysis found that negative mood score on the WHO-SUBI and social relationship score on the WHO-QOL26 were significantly related to salivary cortisol levels. Our results thus demonstrate that several psychological stress induced by the earthquake was associated with an increase in salivary cortisol levels. These results show similar findings to previous study. We anticipate that this study will provide a better understanding of posttraumatic responses in the early stages of adaptation to the trauma and expand effective prevention strategies and countermeasures for PTSD.

## Introduction

On March 11, 2011, at 2:46 pm, the Higashi-Nihon Earthquake (the Great East Japan Earthquake), the largest disaster to occur in Japan since World War II, hit three prefectures (Miyagi, Iwate, and Fukushima) on the Pacific side of northeastern Japan, with lesser damage in several other prefectures. These regions were severely damaged by the magnitude 9 earthquake and ensuing tsunami. The coastal communities of Kesennuma, Ishinomaki, and Minamisanriku were particularly devastated by the earthquake. More than 20,000 people were killed or went missing as a result of the tsunami and fires after the earthquake, and over 240,000 homes were damaged or destroyed. Immediately after an earthquake, people affected are damaged both physically and emotionally. Even now, the region is fighting intermittent aftershocks.

Over the past dozen years or so there have been several serious disaster events in Japan, including the Hokkaido Nansei-oki Earthquake of 1993, the Great Hanshin-Awaji earthquake of 1995, shocking cases such as the sarin gas attack on the Tokyo subway system in 1995, and so on. Many researchers have studied survivors’ mental health and physical changes after such events [1]–[6]. According to many previous studies of survivors of disaster areas, prevalence of posttraumatic stress disorder (PTSD) ranging from approximately 5 percent to 60 percent is seen in the first 1–2 years after a disaster [7], [8]. Further, more than 60 percent of survivors of disasters are at high risk of PTSD [9]. Fujimori and Fujimori (1996) have researched the mental health of survivors of the Hokkaido Nansei-oki Earthquake and pointed out that survivors can be in critical psychological condition six month after such a disaster [6]. Thus, it has been pointed out that mental health problems of survivors are most evident a certain amount of time after a disasters [10].

Previous studies have reported that based on individual difference and the type of disaster, the rate of psychiatric disorders among survivors either decreased after the second year or was prolonged and became chronic [5], [6], [11]–[13]. There are growing concerns about the development of PTSD in people from the disaster-affected areas. In the Great Hanshin-Awaji Earthquake in 1995, many survivors were injured in mind and body and had mental health problems [9]. Even now, some survivors of this earthquake suffer some sort of trauma.

With regard to the relationship between stress and the body, Cannon (1929) and Selye (1956) provide the foundation for the current interest in this physiological phenomenon [14]–[15]. In addition, McEwen and Stellar suggests that chronic stress responses involve actual physiological changes to body systems and organs, and considerable attention has been paid to acute physiological stress responses and how they might possibly lead to subsequent chronic stress responses [16]. Other previous studies suggest that PTSD is associated with behavioral and physiological pathology, which includes disruption of the hypothalamic–pituitary–adrenal (HPA) axis [15], [17]. The HPA axis is involved in mediating physiological responses to stress and the secretion of the stress hormone cortisol [18]. Cortisol is considered an indicator of psychological and physiological stress and can be used in examining the pathophysiology of PTSD [19]. Some previous studies on cortisol data in PTSD report that cortisol level are high when people feel heavily stressed, a symptom of PTSD that can result from events including but not limited to earthquakes, war, accidents, abuse, or radioactive damage [20]–[22]. During the Hanshin-Awaji earthquake, people who had severe PTSD were found to have significantly higher cortisol levels [2]. Cortisol can be extracted from blood, urine, and saliva. Sampling by saliva collection has attracted attention for being a less stressful and invasive method for estimating cortisol levels than other methods of extraction [23]–[26]. Salivary cortisol level have been reported to reflect unbound forms of blood cortisol in particular, and a very high correlation has been reported between plasma and salivary cortisol levels [25]–[27]. To our knowledge, no study has yet examined psychological and physiological changes in survivors before and after the Great East Japan Earthquake.

In this study, we examined that these changes by collecting samples before the earthquake and three months after the earthquake. We hypothesized that psychological measures regarding the mental health of survivors, such as depression, anxiety would worsen and the saliva cortisol level of survivors would increase, three months after the earthquake, compared to before the earthquake. Our hypothesis was based on the previous studies mentioned above.

## Methods

### Ethics Statement

Written informed consent was obtained from each subject, in accordance with the Declaration of Helsinki (1991). This study was approved by the Ethics Committee of Tohoku University.

### Participants

The study was performed three months after the Great East Japan Earthquake occurred. A total of 14 participants (men: 7; women: 7; age range: 19–26) were recruited from among undergraduate and postgraduate students at Tohoku University. All participants experienced the Great East Japan Earthquake. They live in Sendai and the surrounding areas, including the areas closest to the epicenter of the earthquake. All participants had participated in past psychological experiments and saliva cortisol experiments conducted in our laboratory, and had undergone psychological tests and saliva cortisol tests within the past six months and before the earthquake (experimental period: February 8 to March 7, 2011). All participants were screened for absence of neuropsychiatric disorders using the Mini-International Neuropsychiatric Interview (MINI) [28], [29] and all participants were also interviewed by trained psychologists (initials: AO, NA, NS, and YW) using the Clinician-Administered PTSD Scale (CAPS) [30], [31], a structured interview for screening for posttraumatic stress symptoms. CAPS generally requires that the interviewer undergo a psychiatric diagnostic interview. In accordance with the MINI, no participant was diagnosed as having PTSD. Of the 14 participants, each filled more than one but not all the criteria of three clusters of PTSD symptoms, including re-experiencing of the event, avoidance, and hyperarousal.

### Psychological Measures

To assess any change in quality of life, happiness, and mental health of participants from before to after the earthquake, we administered the following questionnaires to participants.

#### World Health Organization Quality of Life 26 (WHO-QOL26)

The World Health Organization Quality of Life 26 (WHO-QOL26) is a 26-item self-report measure designed to assess quality of life (QOL). Twenty-four items measure the four domains of QOL–physical, psychological, social, and environmental–and the other two items measure overall QOL and general health. The score for each question ranges from 1 to 5; higher scores reflect higher QOL. This study used the Japanese version of the WHO-QOL26, which was created by Tasaki and Nakane (1997) [32].

#### World Health Organization Subjective Well-being Inventory (WHO-SUBI)

The World Health Organization Subjective Well-being Inventory (WHO-SUBI) is a 41-item self-report measure designed to assess subjective well-being [33], [34]. The score for each question ranges from 1 to 3. The WHO-SUBI measures two types of subjective well-being. One is “positive affect,” which is measured by the indices of good psychological health (19 items), while the other is “negative affect,” which is measured by the indices of poor psychological health (21 items). Thus, the test evaluates the positive and negative aspects of 11 factors: sense of satisfaction, sense of achievement, self-confidence, sense of happiness, support of close relatives, social support, family relationships, sense of spiritual control, sense of physical ill health, and dissatisfaction with social ties. The reliability and validity of the Japanese version have been demonstrated by Ono et al. [35], [36].

#### General Health Questionnaire (GHQ30)

The General Health Questionnaire (GHQ30) is self-report measure designed to assess underlying psychological distress. This questionnaire comprises 30 questions covering a range of neurotic symptoms, with an emphasis on those typical of anxiety and depression and with a deliberate avoidance of those that might also reflect physical illness [37]. The responses are made on a four-point ordinal scale. The response for each item is scored from 0 to 3 and then summed over the 30 items (range 0–90). This study used the Japanese version of the GHQ30, which was created by Nakagawa and Daibo (1996) [38].

#### Profile of Mood States (POMS)

The Profile of Mood States (POMS) is a 65-item self-report measure designed to assess seven aspects of mood (anxiety/tension, depression/dejection, anger/hostility, confusion/bewilderment, vigor/activity, fatigue/inertia, and friendship). Factor analyses by the developers failed to confirm the friendship domain, and although the guidelines for administration that were followed in this trial continue to include the seven friendship items, they are no longer reported as a POMS subscale or included in the TMD score [39]. Responses to each item range from 0 to 4, with higher scores indicating a more negative mood. This study used the Japanese version of the POMS, which was created by Yokoyama and Araki (1994) [40].

#### Cornell Medical Index (CMI)

The Cornell Medical Index (CMI) consists of 18 sections and 195 items. The A–L sections (144 items) represent physical state and the M–R sections (51 items) represent mental state. Participants answered “yes” or “no” to indicate the presence or absence of a symptom or disorder. If the answer was “yes,” it indicated that the patient had symptoms and received a score of 2. On the other hand, a “no” answer indicated that the patient had no symptoms and was scored at one point [41]. This study used the Japanese version of the CMI, which was created by Kanehisa and Fukamachi (1972) [42].

#### Saliva sampling

We collected saliva samples from participants to measure salivary cortisol level. In consideration of the circadian cortisol rhythms of the participants, we collected saliva samples across the board at 5 pm on weekdays, both pre-examination (before the earthquake) and post-examinations (after the earthquake). The reason we selected 5 pm is because people at this time of day are less affected by circadian cortisol rhythms [43] and we wished to consider participants’ experimental spread-over. We also ordered participants to refrain from drinking, eating [44] and exercise [45] for two hours before saliva sampling.

### Sampling Strategy

Saliva samples were collected using the salivette (Sarstedt, Nümbrecht, Germany) and centrifuged at 3,000 rpm for 5 minutes. We stored the supernatant solution in an airtight container at minus 80 degrees and measured cortisol using the solution. We measured the cortisol with a semi-microcolumn High Performance Liquid Chromatography (HPLC) system (Shiseido, Tokyo). For reagents, we used cortisol and cortisone (Nacalai Tesque, Inc., Kyoto).

The acetonitrile and methanol used were those available on the market. As for the standard solution of CS and CZ, the CS and CZ were dissolved in methanol so that 0.1 mg/ml each (CS: 275.9 nmol/ml, CZ: 277.5 nmol/ml) was used as the original liquid; this was then diluted with 100% methanol or 10% methanol (for CS and CZ, respectively) to be the standard solution used. As for the semi-microcolumns, we used the Capcell Pak MF Ph-1 (4.6 micrometer i.d. times 50 mm) from Shiseido Co., Ltd. as the semi-microcolumn for the preprocessing column, Capcell Pak C18 UG120 (1.5 micrometer i.d. times 250 mm) of Shiseido Co., Ltd. as the semi-microcolumn for the analytical column, and Capcell Pak C18 UG120 (2.0 micrometer i.d. times 35 mm) of Shiseido Co., Ltd. as the semi-microcolumn for the concentrating column.

For HPLC analysis, we set the following conditions: the mobile phase for preprocessing used 5 millimoles per liter phosphoric acid buffer solution (pH = 6.9) and acetonitrile at a ratio of 98/2, and the solution was sent at the flow rate of 1 ml/min. The mobile phase for measurements used 10 millimoles per liter phosphoric acid buffer solution (pH = 6.9) and acetonitrile at a ratio of 78/22, and the solution was sent at the flow rate of 0.1 ml/min. The column temperature was kept constant at 35 degrees Celsius with the detection wavelength set at 242 nm. The duration of salivary cortisol levels was 50 min per sample.

### Experimental Procedure

In this study, participants were measured within subject, pretest (before the earthquake) and posttest (after the earthquake). Both of these experiments followed the same procedure. Participants were tested in the lab of university at weekday afternoon. Participants received experimental suggestions from experimenter and were answered all psychological measures prepared by experimenters in the time given. Participants were allowed 60 min as response time to psychological measures. Then, participants took a saliva sampling at 5 pm. Saliva sampling time was for about five minute. Total experimental time was 65 minutes.

### Statistical Analyses

The statistical analyses of psychological and salivary cortisol data between participants the high PTSD score group and the low PTSD score group were analyzed using the statistical software PASW Statistics 18 for Windows (SPSS Inc, Chicago). Pre- and post-levels of measured variables were analyzed by a paired t-test, and exacerbating factors in salivary cortisol were analyzed by multiple regression analysis. We set the significance level at *p*<0.05.

## Results

First, we examined sex differences in salivary cortisol level, because several previous studies have indicated that it shows a sex difference in stress response [46]–[48]. The result showed no significant differences between males and females (Table 1). We thought that males and females might not differ in the face of the statistics, so we put all of the data together and analyzed it.

**Table 1:** Sex differences of the participants.

	Men (N = 7)	Women (N = 7)	P value
Before the earthquake(mean±SD)	4.3529±2.8122	4.153±2.8992	0.898^a^
After the earthquake(mean±SD)	14.3546±12.6064	9.2219±5.5099	0.343^a^

a : an independent-samples t-test.

Next, the comparison results for the data obtained from these tests are shown in Table 2. First, we examined changes in the scores of the psychological measurements before and after the earthquake. According to the results of the paired t-test, positive scores on the WHO-SUBI, negative scores on the WHO-SUBI, and the social relationship score of the WHO-QOL26 significantly increased (positive score of the WHO-SUBI: *t* = 2.166, degree of freedom [*df*] = 13, *p* = 0.05; negative score of the WHO-SUBI: *t* = 3.183, *df* = 13, *p* = 0.01; social relationship score of the WHO-QOL26: *t* = 2.222, *df* = 13, *p* = 0.05). Emotional status per the CMI and confusion score per the POMS also significantly increased (emotional status: *t* = −2.471, *df* = 13, *p*<0.05; confusion score: *t* = −3.633, *df* = 13, *p*<0.01).

**Table 2:** Characteristics of the participants.

Age [years], (mean±SD)	20.64±2.53		
CAPS Total score, (mean±SD)	17.07±9.38		
	Before theearthquake	After theearthquake	*P value*
The WHO-QOL26 Physical functioning, (mean±SD)	3.07±0.63	2.81±0.38	0.09^b^
The WHO-QOL26 Psychological functioning, (mean±SD)	3.26±0.6	3.27±0.59	0.865^b^
The WHO-QOL26 Social relationship, (mean±SD)	3.67±0.87	3.26±0.83	**0.045** b
The WHO-QOL26 Environmental functioning, (mean±SD)	3.63±0.64	3.46±0.5	0.202^b^
The WHO-QOL26 Global functioning, (mean±SD)	3.54±0.5	3.61±0.86	0.789^b^
The WHO-SUBI positive score, (mean±SD)	41.43±7.71	37.5±5.68	**0.05** b
The WHO-SUBI negative score, (mean±SD)	52.36±6.54	47.14±5.63	**0.007** b
GHQ score, (mean±SD)	5.64±5.88	7.79±6.59	0.375^b^
POMS Tension-Anxiety score, (mean±SD)	53.36±10.4	54.36±12.29	0.77^b^
POMS Depression-Dejection score, (mean±SD)	51.71±10.58	46.0±17.47	0.381^b^
POMS Anger-Hostility score, (mean±SD)	44.57±7.14	47.21±17.26	0.615^b^
POMS Vigour-Activity score, (mean±SD)	50.86±7.42	47.86±15.33	0.577^b^
POMS Fatigue-Inertia score, (mean±SD)	52.64±10.12	53.07±15.83	0.915^b^
POMS Confusion score, (mean±SD)	51.0±9.54	65.14±13.92	**0.003** b
POMS Total Mood Disturbance score, (mean±SD)	202.43±38.84	217.93±29.96	0.3^b^
CMI somatic status score, (mean±SD)	14.57±8.67	18.21±15.74	0.278^b^
CMI emotion status score, (mean±SD)	7.64±6.33	19.14±21.07	**0.028** b
Salivary cortisol level, (mean±SD)	4.25±2.45	11.79±9.72	0.17^b^

b : Paired t-test.

Next, we measured cortisol levels from after the earthquake. The results of the comparison of salivary cortisol level before and after the earthquake are given in Table 2; they show that salivary cortisol level had increased by three months after the earthquake, a difference with statistical significance (*t* = −2.745, *df* = 13, *p*<0.05).

To pinpoint the exacerbating factors in salivary cortisol level, a multiple regression analysis was conducted using the difference between salivary cortisol level before and after the earthquake as the objective variable. The CAPS score, positive score on the WHO-SUBI, negative score on the WHO-SUBI, and the social relationship score of the WHO-QOL26 were used as explanatory variables. The results of the multiple regression analysis are shown in Table 3. It was found that negative scores on the WHO-SUBI and the social relationship score of the WHO-QOL26 were factors significantly related to salivary cortisol level.

**Table 3:** Results of a multiple regression analysis of change in salivary cortisol levels before and after the earthquake.

Explanatory variable	Objective variable	
	Change in salivary cortisol levels before and after the earthquake	
	β	p
CAPS score	1.02	**0.004**
Positive score of the WHO-SUBI	−0.022	0.923
Negative score of the WHO-SUBI	0.717	**0.021**
Social relationship score of the WHO-QOL26	0.653	**0.029**
R^2^	0.696	

* β: Standardized Coefficients.

R^2^ : R Squar.

P: Significance probability.

## Discussion

To investigate psychological changes among the survivors in the disaster area before and three months after the earthquake, we used psychological measures and salivary cortisol level. The results show that salivary cortisol levels increased after the earthquake. The exacerbating factors in this increase were negative scores on the WHO-SUBI and (higher) social relationship score of the WHO-QOL26. Our findings were consistent with the results of previous studies, such as those on the Hanshin-Awaji earthquake of 1995 and the Wenchuan earthquake of 2008 which were associated with higher cortisol level [2], [4]. The relationship between higher cortisol levels and psychological stress suggests that high stress conditions induce an alteration in the HPA axis and stimulate the release of cortisol [49]–[51].

Some exacerbating factors in salivary cortisol level may have included anxiety as a result of the disruption of access to daily information, damage to homes or businesses, and lifeline damage such as interruption of water supply, gas supply, or electrical power supply. Another possible factor was logistical disruption and resultant shortages of food, drinking water, and gasoline. Many people who were in the disaster still spend their days feeling stress, fear, fatigue, helplessness, disappointment [11], and so on. It would appear that these factors still influence psychological responses to the disaster three months after the earthquake. Previous studies have suggested that psychological responses in times of disaster can be categorized into three steps: “Reactions,” “Factors,” and “Psychodynamics” [52], [53]. “Reactions” are the psychological states associated with the stress situation, “Factors” are the various kinds of circumstance said to contribute to the reactions observed, and “Psychodynamics” are the character and mode of operation of the alleged relationships between the normal or abnormal reactions observed and the factors contributing to them [52]. The current situation in the disaster area is in transition from Factors to Psychodynamics. There are cases of spontaneous psychological recovery; however, many survivors still have strong negative feelings such as depression, anger, and disconcertment around the earthquake. It would appear that living conditions changed dramatically soon after the earthquake disaster because of temporary living in evacuation centers, the inconvenience of getting things, lack of essential utilities, etc. Moreover, as people had to live in an environment that was complex and unfamiliar, it is considered that they felt more anxiety and fatigue than usual in their daily living and human relationship [54]. With these points in mind, we consider that the altered daily life environment and psychological stress caused by the disaster influenced an increase in salivary cortisol level. In summary, our study demonstrated that the severe psychological stress induced by the Great East Japan Earthquake was associated with salivary cortisol level and that psychological changes resulted from this earthquake, although the difference was not significant.

In the way of limitations of this study, four points should be noted. 1) The sample size of this study was very small. This is because the psychological and saliva cortisol data from before the earthquake were conducted for the purpose of collecting baseline data for other preliminary experiments, and the number of participants in the preliminary experiment was 14. This study compared the data of these 14 participants before the earthquake and three months after the earthquake. 2) We could not consider gender-differentiated salivary cortisol levels or psychological measures, because the numbers of men and women in participants was very small and because the salivary cortisol levels of participants showed no significant difference by gender. 3) Because this study compared data before the earthquake three months after the earthquake, we did not use new depression scales such as the Center for Epidemiologic Studies Depression scale (CES-D) or the Self-rating Depression Scale (SDS). 4) We did not check or consider coping behavior or resilience of survivors, for the same reason as in point 3.

In the future, we plan to examine the following three points: 1) First, we will examine change in activity of natural killer cells before and after the earthquake, because these cells are associated with cortisol levels and immune activity in stress conditions [55]. We already have blood samples from participants, obtained before and three months after the earthquake. Therefore, we will immediately analyze the change in activity of natural killer cells in the blood. 2) Second, we will examine the same variables in the same participants six months, twelve months, or three years after the earthquake, as a follow-up study. Some previous studies about earthquakes have done follow-ups a few years later [56]–[59]. We consider it very important for the mental health of survivors to track psychological changes and salivary cortisol levels over time. 3) As an additional experiment, we are considering studying survivors living in the coastal area devastated by the earthquake. This study was conducted with survivors living in Sendai and the surrounding, areas which were less affected by the earthquake. The reason we were unable to study coastal survivors was that we did not have psychological and saliva data from them before the earthquake. However, time has passed since the Great East Japan Earthquake, and we think that it is important to conduct a longitudinal study of the mental health of survivors living in the area most severely affected by the earthquake. And not only that, we need to consider the importance of examining the new lifestyles and psychological support for survivors based on the outcomes of studies. To gather further data, we feel that a cohort study of these individuals is important; although we will not have data from before the earthquake to fall back on, we will be able to assess changes over time using the longitudinal cohort model. Therefore, we want to examine these issues after improvements of the methodology used in this study.

In conclusion, more than half a year after the earthquake, rebuilding efforts in the disaster area are very slow. Many survivors still have to confront painful situations such as life stress after the earthquake, disparity between survivors in the disaster area, employment problems, and the effect of radiation. Especially in the worst-affected coastal area, survivors have become extremely unstable. Our overarching mission is to assess about change over time in physical and mental health of many people damaged by earthquake using scientific methods.
